# 27.12 MHz Radiofrequency Ablation for Benign Cutaneous Lesions

**DOI:** 10.1155/2016/6016943

**Published:** 2016-04-05

**Authors:** Dong Hyun Kim, Dong Ju Hyun, Raymonde Piquette, Clément Beaumont, Lucie Germain, Danielle Larouche

**Affiliations:** ^1^Centre de Recherche en Organogénèse Expérimentale de l'Université Laval/LOEX, Centre de Recherche du CHU de Québec-Université Laval, Axe Médecine Régénératrice, and Département de Chirurgie, Faculté de Médecine, Université Laval, 1401 18ième rue, Québec, QC, Canada G1J 1Z4; ^2^Department of Dermatology, CHA Bundang Medical Center, CHA University, 59 Yatap-ro, Bundang-gu, Seongnam-si, Gyeonggi-do 463-712, Republic of Korea; ^3^Dectro International, 1000 boulevard du Parc Technologique, Québec, QC, Canada G1P 4P1

## Abstract

As surgical and/or ablative modalities, radiofrequency (RF) has been known to produce good clinical outcomes in dermatology. Recently, 27.12 MHz RF has been introduced and has several advantages over conventional 4 or 6 MHz in terms of the precise ablation and lesser pain perception. We aimed to evaluate the clinical efficacy and safety of 27.12 MHz RF for the treatment of benign cutaneous lesions. Twenty female patient subjects were enrolled. Digital photography and a USB microscope camera were used to monitor the clinical results before one session of treatment with 27.12 MHz RF and after 1 and 3 weeks. Treated lesions included telangiectasias, cherry and spider angiomas, skin tags, seborrheic keratoses, lentigo, milium, dilated pore, acne, piercing hole, and one case of neurofibroma. For vascular lesions, clinical results were excellent for 33.3%, good for 44.4%, moderate for 11.1%, and poor for 11.1%. For nonvascular lesions (epidermal lesions and other benign cutaneous lesions), clinical results were excellent for 48.3%, good for 45.2%, moderate for 3.2%, and poor for 3.2%. No serious adverse events were observed. Mild adverse events reported were slight erythema, scale, and crust. The 27.12 MHz RF treatment of benign vascular and nonvascular lesions appears safe and effective after 3 weeks of follow-up.

## 1. Introduction

Electrosurgery is defined as the use of heat generated in body tissue through tissue resistance to high-frequency alternating current for the destruction and removal of diseased tissue or for cutting through normal tissue with minimal bleeding [1]. Since electrosurgery had been introduced in dermatologic practice in the 1950s, it became a dermatologic treatment for skin cancer [2]. Although electrosurgery for skin cancer was replaced by Mohs micrographic surgery, it continues to be used routinely for treatment of a variety of benign dermatologic conditions and for epilation, the process of removing unwanted hairs [3, 4].

Radiofrequency (RF) delivers rapidly alternating currents from a probe tip to tissue, which produces a thermal effect on target by resistive heating or impedance to current flow [5]. Thermal effect of RF current on tissue is dependent on both the electrical properties of the tissue and the electrode configuration [6]. During RF procedure, temperature increases over 60°C (70–90°C) at electrode-tissue interface causing collateral thermal damage. To achieve minimal scarring, RF device should have high-frequency power with low intensity, tissue heating during the procedure should be minimal, and the diameter of electrode in contact with the tissue should be as small as possible [7, 8].

The range of designated frequencies used for RF in dermatology has been increasing since 1925 from 0.5 MHz to 1.7 MHz to 3.35 MHz to 4 MHz. Then, in 1985, the FCC (The Federal Communications Commission) introduced the ISM rules for industrial, scientific, and medical applications with frequencies of 6.758 MHz, 13.56 MHz, and 27.12 MHz [9].

Previously, we had conducted the histological evaluation of thermal effect of 27.12 MHz RF on ex vivo human hair follicle tissue. The results showed that thermal damage extended over several hundred micrometers (100–400 *μ*m) from the center of the inserted probe [4]. Although 27.12 MHz RF has been studied for hair removal, there is no clinical study of the treatment for benign vascular and epidermal lesions. In this study, we evaluated the clinical efficacy and safety of a first 27.12 MHz RF treatment to ablate benign vascular and epidermal lesions.

## 2. Materials and Methods

### 2.1. Study Design and Study Population

This pilot study was approved by the “comité d'éthique de la recherche du Centre de Recherche du CHU de Québec-Université Laval” for the protection of human subjects.

The study subjects were patients presenting benign cutaneous lesions treated at Académie Dectro. Patients who had undergone concomitant treatments for their benign cutaneous lesions including laser, IPL, and electrosurgery/radiosurgery within the previous 6 months were excluded and not eligible. Patients with keloid, bleeding tendency, nevus, and immunosuppression were also excluded. Thus, 20 patients were enrolled after obtaining a written informed consent for participation in this research. Patients completed a self-administered questionnaire to collect detailed personal history (skin type, skin symptoms, health condition, and drugs). Mean patient age was 47.2 years old (range 18–72) and all were female with Fitzpatrick skin phototypes from II to IV.

### 2.2. Procedures

Each patient took one session of treatment with a 27.12 MHz RF device (Eclipse, Dectro International, QC, Canada). According to manufacturer's protocol, two operation modes were used: TeleFlashTM mode (pulses in thousandth of a second) and MultiCoagulationTM mode (slow heating followed by TeleFlashTM mode). Peak temperature in the skin at the moment of RF treatment approximates 75°C (167°F) (manufacturer's data). No anesthetic procedure was used during the procedure. Patients were instructed to use a moisturizer (Action de Gala, Dectro International, QC, Canada) several times for a few days after treatment to promote wound healing and to minimize skin dryness.

### 2.3. Objective and Subjective Evaluations

Photographs were taken using identical digital camera (Nikon D3100) and USB microscope (M2, Scalar Corporation, Tokyo, Japan) settings, lighting, and subject positioning at baseline and 1 week and 3 weeks after the treatment. A dermatologist performed the clinical evaluation. The clinical results were scored as excellent (complete reduction), good (more than 75% reduction), moderate (more than 50% reduction), or poor (less than 50% reduction).

The subjects were surveyed during the last follow-up (3 weeks after the treatment) to determine their overall level of satisfaction with treatment results using the following scale: very satisfied, satisfied, slightly satisfied, and unsatisfied. Relative pain scores associated with the treatment were evaluated using 10 cm visual analog scales (VAS), with 0 being “no pain” and 10 being “extremely painful.” The occurrence of adverse events was evaluated at each visit.

## 3. Results

A total of 20 female patients were enrolled and completed the treatment session with a 27.12 MHz RF device. Selected baseline characteristics of the study population are described in Table 1. One patient was lost to follow-up. The total number of treated lesions was 67 in 20 patients (mean 3.3 per patient). There were 36 vascular lesions, including 22 telangiectasias, 13 cherry angiomas, and 1 spider angioma. There were 31 nonvascular lesions (epidermal and other benign cutaneous lesions), mostly epidermal lesions, including 13 skin tags, 8 seborrheic keratoses, 1 lentigo, and 9 others (Table 2).

**Figure 1:**
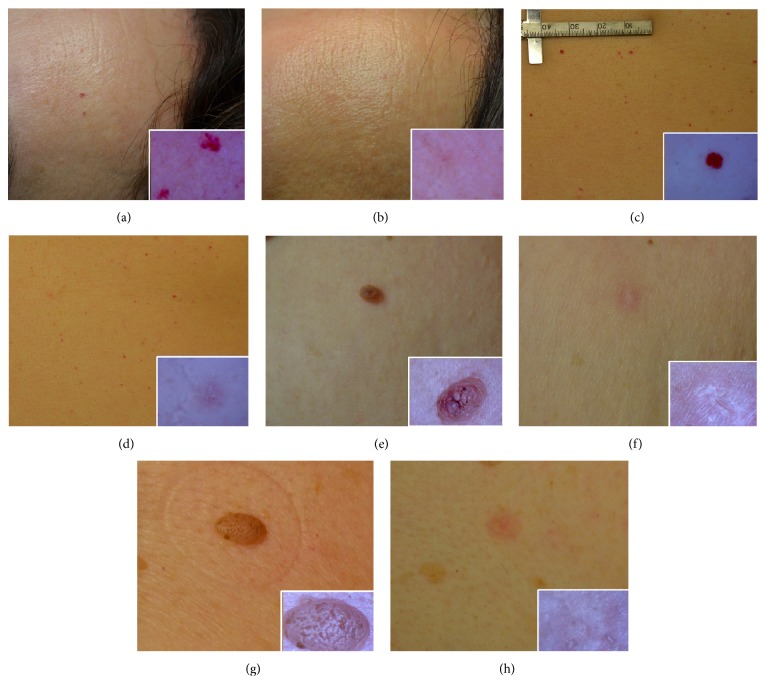
Representative photographs of vascular lesions treated with radiofrequency. Cherry angioma on the forehead at baseline (a) and the end of treatment (b). Cherry angioma on the abdomen at baseline (c) and the end of treatment (d). Skin tag on the axilla at baseline (e) and the end of treatment (f). Seborrheic keratosis on the anterior chest at baseline (g) and the end of treatment (h). Insert: appearance of the lesion under USB microscope.

Representative photographs of positive clinical treatment outcomes for different skin lesion types treated with RF are presented in Figure 1. The clinical treatment outcomes of vascular lesions indicated that 12 cases (33.3%) showed excellent results; 16 cases (44.4%) showed good results; 4 cases (11.1%) showed moderate results; and 4 cases (11.1%) showed poor results (Table 2). Lesions situated on the face showed good response to the treatment. Two of the subjects showing moderate improvement had a medical history of photodamaged skin and rosacea, respectively. The anatomic sites of cases which showed poor results were nasal ala and upper eyelid, which are generally prone to pain. Besides, nasal ala is generally difficult to approach.

The clinical treatment results of nonvascular lesions had similar outcome to those of vascular lesions, which showed 15 cases (48.3%) with excellent, 14 cases (45.2%) with good, 1 case with moderate, and 1 case with poor results (Table 2). The epidermal lesion that showed poor result was a case of lentigo on the lateral canthus area, which remained erythematous at the end point of treatment. Moderate outcome was observed in the case of piercing hole.

Surveys evaluating overall satisfaction with treatment revealed that, for vascular lesions, 34 cases (94.4%) were very satisfied and 2 cases (5.6%) were satisfied. For nonvascular lesions, 26 cases (83.9%) were very satisfied and 5 cases (16.1%) were satisfied (Table 2).

In this study, no anesthesia procedure was used. The treated area usually became erythematous and swollen within several minutes after the treatment. In most patients, minimal discomfort was reported during the RF procedure and it disappeared immediately after. The mean relative pain score using VAS was 3.11 for the treatment of vascular lesions and 3.95 for the nonvascular lesions (Table 2). Subjects reported higher pain score in the pain-sensitive area such as nostril, nasal ala, and eyelid. During the procedure, subjects reported the gradual decrease of pain intensity. Serious adverse events were not reported. As mild adverse events, slight erythema, scale, or crust was found at the end point of treatment in 19 vascular (52.7%) and 19 nonvascular (61.3%) lesions (Table 2).

## 4. Discussion

In the present study, we observed that clinical results following a first 27.12 MHz RF treatment to treat vascular, epidermal, and other benign cutaneous lesions were excellent for 40.3%, good for 44.8%, moderate for 7.4%, and poor for 7.4% after 3 weeks of follow-up. Regarding the patients with moderate and poor results, it is not excluded that a second course of treatment could lead to satisfactory results. However, it is recommended to wait a minimum of three weeks between treatments in order that skin recovers.

Many diverse treatments can be chosen for benign cutaneous lesions, such as surgical excision, CO_2_ laser [10], Er:YAG laser [11], trichloroacetic acid (TCA), and cryosurgery [12] for ablating epidermal lesions. Also, pulsed dye, alexandrite, KTP, and Nd:YAG lasers and intense pulsed light (IPL) can be used for cutaneous vascular lesions [13]. RF ablation is an effective surgical treatment for benign cutaneous lesions. In many countries, RF continues to be the preferred method and has been used along with electrosurgery. RF ablation procedure has been shown to be quick, bloodless, and leading to good cosmetic outcomes [5, 7, 14].

In this study, we evaluated the efficacy and safety of 27.12 MHz RF for the treatment of benign cutaneous lesions. Benign vascular anomalies, especially on exposed sites, are associated with psychological distress [15, 16]. Traditionally, electrosurgery has been used to treat benign skin tumors such as spider angiomas, telangiectasias, and cherry angiomas [17]. In 1983, selective photothermolysis by Anderson and Parrish dramatically transformed the trend of the treatment for cutaneous vascular lesions [18]. Lasers with various wavelengths or IPL are now considered the gold standard treatment for many cutaneous vascular anomalies [19]. These techniques are based on the selective absorption of a brief radiation pulse that generates and confines heat at certain pigment targets and are also good and safe methods to reduce the number and size of hairs [20]. Though vascular lasers proved to be fairly effective, the high cost for devices and maintenance is an obstacle in real practice, whereas RF is inexpensive, is easy to handle, and needs little maintenance. Herein, we observed that 27.12 MHz RF effectively treats various vascular lesions with no serious adverse events. We consider that 3 weeks of follow-up was sufficient to conclude the complete recovery of the skin after the intervention, making a fourth follow-up visit unnecessary for patients. Because the size of treated vascular lesions was less than 1 mm, treatment outcomes were very good. However, thick and deep vessels, for example, located on thighs, were hard to treat, requiring multiple treatment sessions.

Although many therapeutic options exist for acne, relapse often occurs after treatment is stopped. Lee et al. reported that selective sebaceous gland electrothermolysis using a high-frequency electrical current can be a safe and effective method of achieving consistent remission in acne [21]. In our cases, one patient with inflammatory acne on both cheeks and mandible was treated with RF. The number of inflammatory comedones had decreased without any anti-acne treatment three weeks after one session of RF treatment. Because the number of cases is limited, more cases are needed to evaluate the efficacy of RF on acne.

One patient had a dermal neurofibroma on the shoulder. Multiple cutaneous neurofibromas are usually found in individuals with von Recklinghausen disease or neurofibromatosis type 1. Although dermal neurofibromas do not become malignant, neurofibroma with pain and disfiguring features can be treated with surgical excision or CO_2_ lasers. Kim et al. reported multiple cutaneous neurofibromas treated with 4 MHz RF ablation and excision in 16 neurofibromatosis type 1 patients [22]. Although dermal neurofibroma was incompletely removed in our case, the patient was satisfied with the clinical result and the procedure was quick and simple with no bleeding. RF can be an alternative option for the treatment of multiple cutaneous neurofibromas.

RF using 27.12 MHz has several advantages over conventional 4 MHz RF. Because the sensation perceived by the patient seems to be less intense as the frequency increases, a frequency of 27.12 MHz will be better for pain control. Also, higher frequency has a more efficient power absorption rate by water molecules, meaning that less power is needed to destroy the target. Furthermore, the rapid change from positive to negative polarity—27 million cycles per second—allows more precise and fast electrocoagulation.

## 5. Conclusions

RF ablation using 27.12 MHz was very effective and safe for benign vascular and nonvascular cutaneous lesions with minimal adverse events. Most lesions were easily treated with no anesthesia during the procedure. The time for the procedure did not exceed a few minutes for each lesion. All patients rarely complained about pain during the procedure and pain control was not needed. Further clinical trials are needed to clarify its indications for benign cutaneous lesion treatment.

## Figures and Tables

**Table 1:** Characteristics of patients and treated lesions.

Characteristics	Frequency (*n*)	Percentage (%)
Age		
10–29	2	10
30–49	7	35
>50	11	55
Skin type		
II	1	5
III	18	90
IV	1	5
Type of lesion		
Vascular lesion		
Telangiectasia	22	32.8
Cherry angioma	14	20.8
Spider angioma	1	1.5
Epidermal lesion		
Skin tag	13	19.4
Seborrheic keratosis	8	11.9
Lentigo	1	1.5
Miscellaneous		
Milium	2	3.0
Dilated pore	3	4.5
Acne	2	3.0
Piercing hole	1	1.5
Neurofibroma	1	1.5
Location of lesion		
Face & neck		
Periocular area	3	4.5
Nose	12	17.9
Others	24	35.8
Torso		
Back	5	7.5
Chest	8	11.9
Abdomen	7	10.4
Axilla	3	4.5
Extremities		
Upper extremities	1	1.5
Lower extremities	5	7.5

**Table 2:** Clinical improvement and adverse events in response to 27.12 MHz radiofrequency.

Clinical improvement							
Type of lesion	Number of cases	Objective improvement				Overall satisfaction	
		Excellent	Good	Moderate	Poor	Very satisfied	Satisfied
Vascular lesion							
Telangiectasia	22	3	13	4	2	20	2
Cherry angioma	13	8	3	0	2	13	0
Spider angioma	1	1	0	0	0	1	0
*Total*	*36*	*12 (33.3%)*	*16 (44.4%)*	*4 (11.1%)*	*4 (11.1%)*	*34 (94.4%)*	*2 (5.6%)*
Epidermal lesion							
Skin tag	13	10	3	0	0	13	0
Seborrheic keratosis	8	4	4	0	0	8	0
Lentigo	1	0	0	0	1	0	1
Miscellaneous (dilated pore, milium, neurofibroma, acne, and piercing hole)	9	1	7	1	0	5	4
*Total*	*31*	*15 (48.3%)*	*14 (45.2%)*	*1 (3.2%)*	*1 (3.2%)*	*26 (83.9%)*	*5 (16.1%)*

Type of lesion	Adverse events					Pain	
	Serious			Mild^*∗*^ (percentage)		Mean VAS score	

Vascular lesion							
Telangiectasia	None			10 (45.5%)		3.16	
Cherry angioma	None			9 (69.2%)		3.27	
Spider angioma	None			None		0	
*Mean*	*None*			*52.7%*		*3.11*	
Epidermal lesion							
Skin tag	None			7 (53.8%)		4	
Seborrheic keratosis	None			7 (87.5%)		4.19	
Lentigo	None			1 (100%)		3	
Miscellaneous (dilated pore, milium, neurofibroma, acne, and piercing hole)	None			4 (44.4%)		3.78	
*Mean*	*None*			*61.3%*		*3.95*	

Clinical evaluation was performed by a dermatologist. Clinical results were assessed by physical examination, photographic follow-up, and USB microscope (M2, Scalar Corporation, Tokyo, Japan). The results were described as excellent (complete reduction); good (more than 75% reduction), moderate (more than 50% reduction), and poor (less than 50% reduction). VAS: 10 cm visual analog scales. ^*∗*^Adverse events reported were slight erythema, scale, and crust.
